# Clinical and microbiological characterization of *Clostridium difficile* infection in Romania (2013-2014); a hospital-based study

**DOI:** 10.1186/1471-2334-14-S7-O24

**Published:** 2014-10-15

**Authors:** Gabriel Adrian Popescu, Roxana Serban, Adriana Pistol, Andreea Niculcea, Andreea Preda, Daniela Lemeni, Ioana Macovei, Monica Popoiu, Cristina Ţenea, Daniela Tălăpan, Dragoş Florea, Alexandru Rafila

**Affiliations:** 1 Carol Davila University of Medicine and Pharmacy, Bucharest, Romania; 2 National Institute for Infectious Diseases "Prof. Dr. Matei Balş", Bucharest, Romania; 3 National Institute of Public Health, Bucharest, Romania; 4 Cantacuzino National Institute for Research and Development for Microbiology and Immunology, Bucharest, Romania

## Background

Since 2011 *Clostridium difficile* infection (CDI) has been an emerging nosocomial problem in Romanian hospitals, due to its growing incidence and severity. Objectives: To describe risk factors and clinical outcome for CDI cases, and strains characterization.

## Methods

We collected data for all 398 confirmed or probable cases of CDI admitted during 15 November 2013-28 February 2014 in 11 hospitals: 5 from Bucharest, and 7 from Cluj, Iaşi, Timişoara, Târgu Mureş and Braşov. PCR ribotyping was performed at Cantacuzino Institute and E-test (for moxifloxacin and metronidazole) and binary toxin gene identification (PCR) were performed at Matei Balş Institute. The hospitals sent a maximum 20 feces samples for each test.

## Results

Mean age was 63.4 years (range 1-94 years), and sex ratio F:M=1.08:1. For 40 patients, CDI were community-acquired, 12.5% CI95% (9.3%-16.6%), if indeterminate origin cases were excluded; 12 of 13 strains tested from these patients were ribotype 027 and/or binary toxin positive. A post-antibiotic CDI were documented in 346/385 analyzable cases, 89.9% CI95% (86.5%-92.5%); 53.6% of them received medication from at least two different antibiotic groups. The most utilized antibiotics were cephalosporins and quinolones. In 13.1% cases with known history, the CDI episode was a recurrent one CI95% (10.1%-17%); binary toxin was retrieved in all six tested strains from recurrent CDI. A number of 45 episodes were considered severe CDI (25 deaths, 7 intensive care required, 6 colectomies, 7 patients discharged with worsened condition), 12.1% from 371 episodes with identified outcome CI95% (9.2%-15.8%). From 155 tested strains, 122 belonged to 027 ribotype and/or were binary toxin positive, 78.7% CI95% (71.6%-84.4%); the remaining 33 strains belonged to ribotypes 002 (n=4), 014 (n=2), 018 (n=2), 087 (n=2), one isolate each 001, 011, 012, 017, 020, 106 ribotypes and 17 strains were binary toxin negatives. The 027 ribotype and/or binary toxin presence were prevalent in all hospitals, ranging from 69.7% to 100%. In 62 of 84 tested isolates, the MIC for moxifloxacin was greater than 2 mg/L, the epidemiological cut-off value, more frequent in ribotype 027 or binary toxin positive strains, RR=2.02 (1.27; 3.22), p<0.0001.

## Conclusion

The severe CDI episodes were relatively frequent in our study. The main risk factor for CDI was the previous antibiotic treatment, especially with cephalosporins and quinolones. The wide circulation of 027 ribotype could explain the persistence of hospital outbreaks of CDI and the increase of community-acquired CDI episodes. No discrepancies were noticed among participant hospitals.

